# Gaining Respect and Mitigating Risk: A Qualitative Exploration of How New Attendings Navigate Interpersonal Relationships with Recent Resident Peers

**DOI:** 10.5334/pme.1396

**Published:** 2024-10-03

**Authors:** Cameryn C. Garrett, Hannah Robinson, Jacob David, Brian Utz, Michelle B. Azimov

**Affiliations:** 1 Department of Graduate Medical Education at Community Memorial Healthcare in Ventura, California, USA; 2 Internal Medicine Residency Program at Community Memorial Healthcare in Ventura, California, USA; 3 Family Medicine Residency Program at Ventura County Medical Center in Ventura, California, USA; 4 Family Medicine Residency Program at Community Memorial Healthcare in Ventura, California, USA

## Abstract

**Background:**

Physicians moving through training experience changes in personal and professional relationships, which can increase stress, uncertainty, and burnout. Social connection can be an important resource but can introduce complexity and conflict. This study aimed to explore how early-career attendings navigate and manage changing organizational and friendship roles with recent resident peers (near-peers) through this critical transition.

**Methods:**

We conducted a reflexive thematic analysis of interviews with early-career attendings working with near-peers from the same institution where they trained. Twenty three of 27 (85%) eligible attendings from two United States health systems participated in semi-structured interviews between April and June 2023.

**Results:**

Familiarity from working at the same institution where new attendings completed training made it more difficult to command authority. Early-career attendings at times struggled with insecurities about their ability to fulfill their new role and challenges from others in recognizing their new attending identity. These tensions could heighten emotions in the clinical setting and spill over into relationships with residents outside the workplace, impacting social lives and well-being. Early-career attendings engaged in strategies to manage the social realm of their transition with near-peers, including prioritizing their organizational role in the clinical setting and mitigating risks to their professional reputation by creating stronger boundaries between their personal and professional lives.

**Conclusions:**

This study provides new insight into how attendings navigate changing personal and professional relationships with recent resident peers and offers strategies on how to manage the social realm of this liminal transition.

## Introduction

Physicians undergo major transitions as they move through the continuum of medical education. At each stage, physicians face multiple changes, including level of responsibility for clinical decision making, geographic location, and personal and professional relationships. These transitions can lead to increased stress, uncertainty, and burnout [1,2]. Fostering social connection can be important to enhance physician well-being and minimize burnout; friendships and other social connections, inside and outside the workplace, can have a protective effect and help physicians thrive [3,4].

While workplace relationships provide social benefits, they can also introduce complexity and conflict [5,6]. Little medical research to date has examined how new attending physicians navigate changing relationships with residents who were recently their peers, but evidence from the business literature demonstrates the dynamic nature of workplace relationships and highlights how promotions are a major cause of friendship disruption [7,8]. Following promotion, people can experience tension when expectations between their organizational and friendship roles conflict [7,8,9]. Limited research to date examining social elements of the transition from trainee to attending suggests new attendings can experience angst, stress, and trepidation as they navigate conflicts in care management with recent peers, gauge how recent peers will respond to their new teaching role, and struggle with self-doubt as they build their reputation and identity as an attending [10,11]. Residents who become attendings at the same institution where they trained must contend not only with changes to professional identity, but also with new roles as supervisors required to critically evaluate recent peers.

Although trainees don the new title of attending physician overnight, the full transition is not a single moment of change. Rather, new attendings experience a complex, ongoing process of adaptation as they interact with others in different contexts and manage changing interpersonal relationships and an altered sense of identity [12,13]. To promote well-being, new attendings must engage in strategies to manage both expected and unexpected changes in relationships, as they work to maintain or disengage from resident friendships. Understanding the context for and sources of potential relationship conflict as program graduates transition to attendings can offer guidance on how to support the professional development of new attendings. The aim of our study was to explore how early-career attendings navigate and manage personal and professional relationships in the clinical learning environment with those who were recent peers.

## Methods

### Setting and participants

Participants were enrolled in our study from a convenience sample of early-career attending physicians at two urban Southern California health systems in the United States, Community Memorial Healthcare (CMH) and Ventura County Medical Center (VCMC). Both are community health systems; neither sits within a university system. As such, attendings working with residents are in private solo or group practices. CMH has 98 residents across five programs: Family Medicine (FM), Internal Medicine (IM), General Surgery (GS), Orthopaedic Surgery (OS), and Psychiatry. VCMC has 45 FM residents. Residency training in the United States varies in length by specialty and consists of a supervised full-time clinical training program for medical school graduates. Fellowship is additional subspecialty training for fully credentialed physicians who have completed residency. VCMC hosts five fellowship programs and CMH hosts one; each fellowship is one-year in duration.

Among the five most recent graduate classes, at CMH, an average of 16.7% of GS, 17.3% of FM, 30.0% of OS, and 30.8% of the IM class were contracted as attendings following training; at VCMC, an average of 10.7% of the graduating classes became attendings at the institution. At time of recruitment, the new Psychiatry program had no graduates.

CMH and VCMC attendings working with residents from the same institution where they trained were invited to participate in a one-on-one semi-structured interview. To be eligible, they had to have completed their residency within the last five years. Thirty attendings met this requirement. Two of 30 were excluded because they are investigators on the project and one was excluded based on being an IM chief resident as clinical expectations and administrative duties can vary across institutions and we sought to make our results transferable. Of the remaining 27 eligible early-career attendings, 23 (85.2%) participated. We were interested in interviewing medical and surgical physicians, as well as participants with a range of years since graduation, to identify similarities and differences between different disciplines and length of time since training.

### Data collection and analysis

We constructed an interview guide based on review of medical and business literature and piloted the interview with an attending teaching at the institution where they trained. We recruited by email and text message. CG conducted interviews by phone between April and June 2023. Participants were asked about experiences navigating personal and professional relationships with near peers as they transitioned to attendings and any desired support around navigating these relationships. In interviews, we asked participants about experiences with near peer residents and attendings; for this analysis we focused on resident interactions. The average interview length was 36 minutes. Interviews were audio-recorded and transcribed verbatim. We removed small pauses and repetitions to aid readability [14].

We utilized thematic analysis to examine shared patterns of meaning across interviews [14]. Data collection and analysis were conducted concurrently. We approached our analysis with an experiential orientation centered around participants’ meanings and understandings. We analyzed data using a six-phase reflexive thematic analysis [14]. Analysis was guided by an inductive, critical realist approach [14,15]. We listened to audio files and read and re-read transcripts to become familiar with the data. We imported transcripts into NVivo 11 (QSR International, Burlington, Massachusetts) for data management and we coded data through multiple readings of transcripts. Given the study’s aim, we coded data at a more semantic level with codes staying close to participants’ explicit meaning [14]. We constructed initial candidate themes, using codes as building blocks to examine shared patterns of meaning. To explore relationships between different candidate themes, we created mindmaps in NVivo to help visualize connections and refine themes. We continued to review, refine, define, and then name main themes, referencing transcripts and coded extracts throughout the process. The final phase involved writing the report. Analysis was led by CG. MA reviewed a subset of transcripts and coded extracts; the collaborative approach to coding allowed for a more robust, reflexive understanding and interpretation of data [14].

### Team reflexivity

Our research team included multidisciplinary clinicians and an experienced qualitative researcher. Three authors completed residency at the same institution where they work. Two of these physicians were insider researchers as they graduated within the last five years and recently grappled with managing near-peer relationships. We examined how our assumptions, experiences, and expectations impacted our understanding of the project and interpretation of results, including examining how multiple investigators’ own transitions from resident to attending to program leadership influenced the type of research questions asked and how data were interpreted.

Community Memorial Health System Institutional Review Board (2019-HSR002E) and VCMC Institutional Review Board (345) approved the study.

## Results

Twenty three early-career attending physicians participated in an interview. (See Table 1 for participant characteristics.) We identified three key themes: 1) it’s hard to revere the familiar, 2) prioritizing one’s organizational role, and 3) mitigating professionalism risks to one’s reputation.

**Table 1:** Characteristics of the 23 participants.


VARIABLE	n (%)

**Age**	

30 to 34 years	11 (47.8%)

35 to 39 years	10 (43.5%)

40 to 44 years	2 (8.7%)

**Gender identity**	

Male	16 (69.6%)

Female	7 (30.4%)

**Specialty type**	

Medical	16 (69.6%)

Surgical	7 (30.4%)

**Fellow**	

No	19 (82.6%)

Yes	4 (17.4%)

**Time since**	

**graduating from**	

**residency** ^a^	

< 1 year	3 (13.0%)

1 to 2 years	11 (47.8%)

3 to 5 years	9 (39.1%)


^a^Values do not equal 100% due to rounding.

### It’s hard to revere the familiar

Working as attendings at the same institution where they completed training, participants’ pre-existing relationships with residents influenced attending-resident power dynamics. Being a known quantity could lead to challenges in negotiating power. Current residents had witnessed participants’ struggles and vulnerability on their path to becoming an attending. This transparency could undermine the tacit authority underlying the training hierarchy inherent in residency.

‘I’m sure they don’t look at me as much of an authority figure as I would otherwise had I come from somewhere else … I’ve been sick there [at this hospital]. I’ve been healthy there. I’ve been—you know, I’ve had meltdowns in the bathroom whatever so everyone there knows you and knows everything about who you were as a trainee … that can be challenging to be a professional who also is known personally to all these people.’ *(P09)*

The institutional memory of participants as trainees impacted how they were able to construct their image as an attending. Convincing trainees of their mastery of knowledge was harder when residents saw them gain their knowledge.

‘There’s a respect that comes from just like the magic of not knowing how someone got their knowledge … residents that are junior to me like they know how I got here ((laughter)) … so I think the respect is harder to earn in that sense. It’s not just like kinda thrown at you. You have to really earn it, and I think that’s hard.’ *(P10)*

While time-based residency programs graduate residents on a specific date, participants noted that the perceived clinical acumen of graduates may not differ much from residents who were recent peers. A lack of consensus of where new attendings were on the learning continuum could create tension and hinder their ability to exercise authority. Some participants also spoke about their ‘internal struggle’ *(P06)* to set themselves apart from those training directly under them and cope with anxiety about performing as an attending:

‘It was a little bit difficult at first to take on that leadership role because you did not—I didn’t think I was worthy at that time … it was just harder to see myself in that role because just a few months prior to graduation you were equals.’ *(P23)*

As one’s proximity to the resident class decreased, it became easier to gain respect as one’s resident identity began to fade. For surgical participants, the longer five-year training period compared with three-year medical residencies and the common practice of completing a fellowship elsewhere offered an added degree of separation, which helped them command authority. Returning after fellowship also imbued one with the authority of a content expert and offered a fast track to respect.

Across specialties, women were more likely than men to report experiences where former peers did not fully recognize their new authority. As one participant explained:

‘The residents that were closest in year to me, none of them—even if I asked them to call me by doctor in clinic—they always used my first name … [it’s] very different from their male colleagues who are in the same position as me and graduated. They’ll still call them by Dr. so-and-so … it kind of stings like just a little bit.’ *(P19)*

How residents used honorifics in the clinical setting reflected recognition or rejection of changing status in the medical hierarchy.

Additionally, those in transitional fellowship roles were more likely to experience challenges negotiating power dynamics, such as residents disagreeing with their care plans. Being close friends could intensify the emotional response to disagreements in a workplace encounter and exacerbate dual-role conflict, spilling over to their relationship outside work. As one fellow explained: ‘We had just been on vacation a month ago together and I felt like, “Oh, no, did this one 30-minute conversation undo the whole vacation?”’ *(P10)*. Most fellows shared similar stories of friendships altered temporarily or permanently by disagreements in the enactment of power in the workplace when others did not recognize their attending identity; heightened emotions in some circumstances led to shouting or tears, which could result in ‘a tiny hole’ *(P05)* in the fabric of their friendships.

### Prioritizing one’s organizational role

To help maintain friendships with residents, participants engaged in strategies to manage potential dual-role tensions that could arise when trying to balance their friendship role with their organizational role as attending. The most common strategy in the clinical setting was prioritizing their organization role:

‘I feel like when I’m a physician or a provider rather than a friend in the moment I tend to like place my priorities a little differently and more towards patient safety and patient responsibility … it’s just like there’s that tension of, “No, I’m not a friend right now.” … What’s changed? Well there’s a patient between us.’ *(P10)*

When a patient was present, one’s attending identity took precedence. A few participants specifically sought social support from more established attendings to guide how best to redefine relationships. They were advised to prioritize their attending leadership role by focusing on patient safety and remember that one can’t always *just* be a friend in the clinical setting. This advice mirrors organizational expectations within healthcare institutions that patient care comes first, with institutional goals taking precedence over friendship obligations. The acceptance and positive feedback expected among friends was not always possible in one’s supervisory role [16]. As one moved up the clinical ladder, participants had to re-negotiate their place in the clinical learning environment and re-calibrate friendships.

‘It’s tough when you’re like a new grad and you’re working with people that like knew you as a resident. On one hand it’s like fun ’cause it’s more collegial and so it’s more shared decision-making but I do think it can sometimes make it harder when you want to go in a different direction [clinically] … the big challenge is like making that transition from, hey, I’m not your like peer anymore, I’m kinda the shot caller … [and] having that be well-received can be tricky.’ *(P13)*

Within that context, the hierarchy in medical training was cited as a helpful tool to guide relationship boundaries and reduce ambiguity about which role to privilege in different contexts. Participants noted that the attending’s legal responsibility for the patient helped legitimize deference to attendings in clinical decisions. Prioritizing one’s formal organizational role provided participants and resident friends with a script for how to navigate changes in power dynamics during clinical care.

### Mitigating professionalism risks to one’s reputation

As participants transitioned to attendings, social patterns with residents shifted. While at times altered social interactions happened naturally as attendings with young families prioritized family time, becoming an attending often led participants to take distinct steps to disengage from the wider resident cohort to mitigate professionalism risks. The main strategies utilized to create stronger boundaries between their personal and professional lives were: 1) reducing the flow of certain types of personal information and 2) altering socialization patterns outside the workplace. As participants re-negotiated interactions in social spaces, a common tactic was shifting more of the socializing with residents to group settings. Selecting group activities, such as potluck dinners or hikes, helped foster social connection in the program with a lower risk of being perceived as ‘showing favoritism’ *(P08)*.

Among participants who preferred a stronger boundary between their personal and professional lives as an attending, familiarity could disrupt boundary setting. Past disclosures made it harder to manage personal information circulating in the workplace, which could have reputational consequences:

‘[When you start residency] you kinda share too much at work ((chuckle)). And then you get further along and people know too much about you and you’re like, “Dang, I kinda wish ((chuckle)) these people didn’t know that.” … Those things you overshared can sometimes be a detriment to your authority ((laughter)), and so you share less of those.’ *(P13)*

Participants also had to contend with the permeability of boundaries resident friends set. Some participants wrestled with whether information gleaned from social media or in-person interactions was pertinent to residents’ clinical care. For example, if one knew a resident was late for rounds because they flew in from vacation early in the morning or of occasional substance use, they had to consider whether they were obligated to disclose and act upon that knowledge in a way that challenged friendship expectations.

Although residency programs’ cultures differed in the degree to which socializing with residents outside of the hospital was normative, a higher proportion of female participants compared with males discussed actively mitigating professionalism risks to their reputation by setting stricter professionalism boundaries both inside and outside work. One participant shared how she perceived her concerns about socializing with residents contrasted with male colleagues:

‘If he’s an attending and he wants to take a bunch of people out to go get pizza and beer, he can. And it’s me—it’s like, “Well, I don’t know. Can I do that?” Like I can’t—I don’t wanna show favoritism. I don’t ever want anything I say or do to be misconstrued.’ *(P19)*

Female participants more closely scrutinized social interactions considered commonplace during residency, concerned about how others may judge them. Another woman outlined steps to explicitly signal the change in relationships, such as using formal titles, and cautioned others, ‘you just have to be careful what can be misconstrued’ *(P06)*. To many female participants, the specter of misperception remained a looming threat.

While stronger professional boundaries could help participants navigate the transition, they also had the potential to negatively impact wellbeing, as one fellow’s experience exemplified:

‘Our fellowship has been lonely in a sense that I didn’t wanna upset things or complicate things—muddy the waters at work, so I did pull back a little bit emotionally and … [have] foregone a lot of [social] events.’ *(P05)*

This participant’s experience highlights how efforts to mitigate professionalism risks could conversely negatively impact workplace social support structures. Notably, stronger professional boundaries were most commonly employed by participants towards resident relationships with moderate or low levels of self-disclosure and trust; changes were less common among close friends within which a strong social support network already existed.

## Discussion

This study on early-career attendings’ experiences provides new insight into how attendings navigate changing personal and professional relationships with recent resident peers and offers strategies on how to manage the social realm of this liminal transition. The familiarity that comes from working at the same institution where they completed training made it more difficult for new attendings to command the authority normally inherent in their supervisory role. Early-career attendings at times struggled with insecurity about their ability to fulfill their new role as well as challenges in others recognizing their new attending identity. These tensions could heighten emotions in the clinical setting and spill over into relationships with residents outside the workplace, impacting social lives and well-being. Strategies to navigate the transition included prioritizing their organizational role over friendship role in the clinical setting and mitigating risks to their professional reputation by creating stronger boundaries between their personal and professional lives.

Female participants and fellows were more likely to report workplace tensions around negotiating changing power dynamics. Experiences of female participants align with findings across the medical education continuum that women are more likely to report others questioning their medical decision making and authority [17,18]. Tensions experienced by fellows are likely multidimensional. Their position may not be perceived as one of authority as in some contexts they are still trainees. As very recent residency graduates, they may be more enmeshed with resident peers; close familiarity may prevent them from being viewed as an authority figure. Residents may perceive little distinction between their clinical knowledge and the fellow’s, as the temporal marker of graduation may not highlight a notable difference in clinical acumen from senior residents. When receiving feedback and clinical guidance, residents and attendings engage in a social negotiation situated within the context of their relationship; a key factor in this negotiation is the evaluator’s clinical credibility [19]. Fellows were more likely to have their clinical credibility questioned and to have their authority perceived as not yet legitimate. Even those not in fellowship noted, however, that it could be harder to garner respect and credibility among those who had known them during training when their vulnerabilities and acquisition of knowledge were in view.

Prioritizing one’s formal organization role over one’s friendship role provided a script for attendings on how to interact with resident friends during clinical care. This strategy of prioritizing one domain over another, also defined as selection, is a frequent tactic in organizational settings outside medicine to address dual-role tensions in hierarchical friendships [7,9]. In medicine, the goal of patient safety can help reduce ambiguity around which role should take precedence. Where there are concerns about physician performance impacting patient care, the professional mandate to prioritize patient interests, including patient safety, over personal interest could lead to ethical dilemmas between personal loyalties to resident friends and obligations to patients and profession [20]. These tensions may be heightened as attendings adjust to their new role.

Although constructive effects of the hierarchy are less often examined in medical education [21], participants highlighted how hierarchy was helpful to negotiate social power in the clinical setting and reinforce their new identity by outlining expected organizational roles and responsibilities for resident-attending interactions [22]. By clarifying who was ultimately responsible for patient care, the hierarchy could reduce interpersonal conflict [23]. Outside the clinical setting, no similar formal structure exists to provide a clear script for social interactions. Attendings had to navigate how their promotion altered professionalism expectations in the social domain. Drawing on work around boundary management [24], we found that promotion to attending required making decisions anew around the degree to which participants delineated or blended personal and professional domains in interactions with residents. Most attendings took steps to actively construct stronger boundaries, particularly in friendships with moderate or low levels of self-disclosure or trust, by reducing the flow of personal information and shifting socialization patterns outside the workplace. Notably, professionalism mitigation requires that attendings examine their relationships holistically and consider how they may be inadvertently negatively impacting others [5,25]; residents less closely socially linked to new attendings may feel excluded and perceive favoritism among their peers. Female participants across specialties in our study were more likely to be vigilant in how their relationships could be interpreted by others. Female physicians may be primed to be more cognizant about potential perceived lapses in professionalism and better guard against reputational risks as they report experiencing gendered expectations of what constitutes professional behavior and are subjected to greater scrutiny over their actions [26,27].

Sawatsky et al. [28] have called for more focus in medical education on relationship-building and connectedness. While friendships can increase complexities in the workplace, positive, healthy relationships offer many benefits, including helping physicians process clinical experiences, cope with stress, and reduce burnout [3,29]. Supportive relationships have also been shown to enhance receptiveness to feedback in near peer teaching and can improve learning [28,30]. Given the beneficial impact of friendships, creating stronger boundaries with residents has the potential to harm if too much emphasis is placed on professional boundaries at the expense of disengaging from valuable support structures. We were particularly interested in examining experiences of early-career attendings who trained with residents as they already have longitudinal relationships with learners. Cultivating healthy relationships with trainees is an ongoing negotiation as what is considered appropriate behavior with residents shifts as one moves from resident to attending. Examining these relationships can help clarify elements that can facilitate or hinder healthy, meaningful relationships among trainees and supervisors to help guide physicians as they navigate the transition to attending.

In this study, we used a sample from two health systems in a single Southern California community in the United States; this contextualization may impact the transferability of findings. Some strategies shared by participants to navigate changing personal and professional boundaries may be context specific. For example, the use of the honorific “Doctor” in the clinical setting is standard practice in the United States to signal respect and expertise in a professional context, particularly in the presence of patients [31], and in this cultural setting, honorifics functioned as a linguistic cue to signal professional boundaries. Cultural norms around respect and deference towards authority could impact the degree to which attendings experience challenges with near peers in navigating their new role [32].

The majority of training took place in a relatively small city of about 110,000. As a result, it was common for residents and attendings to interact frequently in the workplace and at times outside work, even when unplanned. Some participants noted that residents they supervised had children the same age as their own children, and their children ended up spending time together in recreational settings. These different interactions may have led to additional tensions among participants around managing personal and professional boundaries and increased concerns about professional reputation risks, which may be less common in larger training sites and cities. Future research could examine how family social interactions between attendings and residents outside of the clinical setting impact boundary management. Given the continually changing technological landscape, future studies could also examine how social media communication and self-disclosure may enhance or mitigate tensions in near peer relationships.

## Conclusion

In summary, this study highlights challenges early-career attendings face in personal and professional relationships as they transition to a teaching role. Our results can inform program-wide steps and actions by individual attendings to help facilitate a smooth transition to becoming an attending.

## Data Accessibility Statement

The dataset is available from the corresponding author on reasonable request and with approval from the Community Memorial Health System Institutional Review Board and the VCMC Institutional Review Board.

## Abbreviations

CMH - Community Memorial Healthcare

FM - Family Medicine

GS - General Surgery

IM - Internal Medicine

OS - Orthopaedic Surgery

VCMC - Ventura County Medical Center

## References

[1] Herchline D, Cohen ME, Ambrose M, Hwang J, Kaminstein D, Kilberg M, et al. Into the unknown: characterizing fellow uncertainty during the transition to unsupervised practice. J Grad Med Educ. 2023; 15(2): 201–8. DOI: 10.4300/JGME-D-22-00221.1 37139214 PMC10150825

[2] West CP, Dyrbye LN, Erwin PJ, Shanafelt TD. Interventions to prevent and reduce physician burnout: a systematic review and meta-analysis. Lancet. 2016; 388(10057): 2272–81. DOI: 10.1016/S0140-6736(16)31279-X 27692469

[3] Corgan S, Ford Winkel A, Sugarman R, Young JQ. From burnout to wholehearted engagement: a qualitative exploration of psychiatry residents’ experience of stress. Acad Med. 2021; 96(5): 709–17. DOI: 10.1097/ACM.0000000000003912 33410608

[4] Kase J, Doolittle B. Job and life satisfaction among emergency physicians: a qualitative study. PLoS One. 2023; 18(2): e0279425. DOI: 10.1371/journal.pone.0279425.r010 36827313 PMC9955602

[5] Pillemer J, Rothbard NP. Friends without benefits: understanding the dark sides of workplace friendship. Acad Manage Rev. 2018; 43(4): 635–60. DOI: 10.5465/amr.2016.0309

[6] Winkel AF, Honart AW, Robinson A, Jones AA, Squires A. Thriving in scrubs: a qualitative study of resident resilience. Reprod Health. 2018; 15(1): 53. DOI: 10.1186/s12978-018-0489-4 29587793 PMC5869777

[7] Horan SM, Chory RM, Craw ES, Jones HE. Blended work/life relationships: organizational communication involving workplace peers, friends, and lovers. Communication Research Trends. 2021; 40(2): 3–47.

[8] Sias PM, Heath RG, Perry T, Silva D, Fix B. Narratives of workplace friendship deterioration. J Soc Pers Relat. 2004; 21(3): 321–40. DOI: 10.1177/0265407504042835

[9] Bridge K, Baxter LA. Blended relationships: friends as work associates. West J Commun. 1992; 56(3): 200–25. DOI: 10.1080/10570319209374414

[10] Allen BR. Transition to practice: from resident to faculty at the same institution. J Grad Med Educ. 2014; 6(4): 799–800. DOI: 10.4300/JGME-D-14-00266.1 26140141 PMC4477588

[11] de Montbrun S, Patel P, Mobilio MH, Moulton CA. Am I cut out for this? Transitioning from surgical trainee to attending. J Surg Educ. 2018; 75(3): 606–12. DOI: 10.1016/j.jsurg.2017.09.034 29055743

[12] Gordon L, Jindal-Snape D, Morrison J, Muldoon J, Needham G, Siebert S, et al. Multiple and multidimensional transitions from trainee to trained doctor: a qualitative longitudinal study in the UK. BMJ Open. 2017; 7(11): e018583. DOI: 10.1136/bmjopen-2017-018583 PMC5719319 29196486

[13] Jindal-Snape D. A-Z of transitions. London: Palgrave; 2016. DOI: 10.1057/978-1-137-52827-8

[14] Braun V, Clarke V. Thematic analysis: a practical guide. London: SAGE Publications Ltd; 2022. DOI: 10.53841/bpsqmip.2022.1.33.46

[15] Willig C. Introducing qualitative research in psychology. 3rd ed. Maidenhead: Open University Press; 2013.

[16] Sias PM, Gallagher E. Developing, maintaining and disengaging from workplace friendships. In: Morrison RL, Wright SL, editors. Friends and enemies in organizations: A work psychology perspective. Basingstoke: Palgrave Macmillan; 2009. pp. 78–100. DOI: 10.1057/9780230248359_5

[17] Billick M, Rassos J, Ginsburg S. Dressing the part: gender differences in residents’ experiences of feedback in internal medicine. Acad Med. 2022; 97(3): 406–13. DOI: 10.1097/ACM.0000000000004487 34709203

[18] Vaa Stelling BE, Andersen CA, Suarez DA, et al. Fitting in while standing out: professional identity formation, imposter syndrome, and burnout in early-career faculty physicians. Acad Med. 2023; 98(4): 514–20. DOI: 10.1097/ACM.0000000000005049 36512808

[19] Telio S, Regehr G, Ajjawi R. Feedback and the educational alliance: examining credibility judgements and their consequences. Med Educ. 2016; 50(9): 933–42. DOI: 10.1111/medu.13063 27562893

[20] Glass LL. Where the rubber meets the road: the challenge of reporting colleagues’ boundary violations. AMA J Ethics. 2015; 17(5): 435–40. DOI: 10.1001/journalofethics.2015.17.5.medu1-1505 25986087

[21] Vanstone M, Grierson L. Thinking about social power and hierarchy in medical education. Med Educ. 2022; 56(1): 91–7. DOI: 10.1111/medu.14659 34491582

[22] Looman N, Battal N, Vanstone M. No doctor is an island. Med Educ. 2023; 57(11): 996–8. DOI: 10.1111/medu.15168 37490936

[23] Castanelli DJ, Weller JM, Molloy E, Bearman M. Trust, power and learning in workplace-based assessment: the trainee perspective. Med Educ. 2022; 56(3): 280–91. DOI: 10.1111/medu.14631 34433230 PMC9292503

[24] Rothbard NP, Ollier-Malaterre A. Boundary management. In: Allen TD, Eby LT, editors. The Oxford handbook of work and family. New York: Oxford University Press; 2016. pp. 109–22. DOI: 10.1093/oxfordhb/9780199337538.013.5

[25] Nelson S. The business of friendship: making the most of our relationships where we spend most of our time. HarperCollins Leadership; 2020.

[26] Alexis DA, Kearney MD, Williams JC, Xu C, Higginbotham EJ, Aysola J. Assessment of perceptions of professionalism among faculty, trainees, staff, and students in a large university-based health system. JAMA Netw Open. 2020; 3(11): e2021452. DOI: 10.1001/jamanetworkopen.2020.21452 33226428 PMC7684446

[27] Xun H, Chen J, Sun AH, Jenny HE, Liang F, Steinberg JP. Public perceptions of physician attire and professionalism in the US. JAMA Netw Open. 2021; 4(7): e2117779. DOI: 10.1001/jamanetworkopen.2021.17779 34328503 PMC8325071

[28] Sawatsky AP, Rea JR, Hafdahl LT, et al. From apprenticeship to assembly line: recovering relationships in medical education. J Grad Med Educ. 2023; 15(6): 627–31. DOI: 10.4300/JGME-D-23-00468.1 38045942 PMC10686647

[29] Leep Hunderfund AN, West CP, Rackley SJ, et al. Social support, social isolation, and burnout: cross-sectional study of U.S. residents exploring associations with individual, interpersonal, program, and work-related factors. Acad Med. 2022; 97(8): 1184–94. DOI: 10.1097/ACM.0000000000004709 35442910

[30] Wong SN, Luo CJ, MacDonald G, Hatala R. A qualitative study of medical students’ perceptions of resident feedback. Med Educ. 2022; 56(10): 994–1001. DOI: 10.1111/medu.14847 35639522

[31] Harvey JA, Butterfield RJ, Ochoa SA, Yang YW. Patient use of physicians’ first (given) name in direct patient electronic messaging. JAMA Netw Open. 2022; 5(10): e2234880. DOI: 10.1001/jamanetworkopen.2022.34880 36197668 PMC9535496

[32] Meyer E. Being the boss in Brussels, Boston, and Beijing: if you want to succeed, you’ll need to adapt. Harvard Bus Rev. 2017; 95(4): 70–7.

